# Supplementary material for: Rapamycin Affects the Hippocampal SNARE Complex to Alleviate Cognitive Dysfunction Induced by Surgery in Aged Rats
Source: Brain Sci. 2023 Mar 31;13(4):598. doi: 10.3390/brainsci13040598 (PMC10136734; doi:10.3390/brainsci13040598)
Supplement: Supplementary file 1 [file brainsci-13-00598-s001.zip › brainsci-2084885-supplementary.pdf]

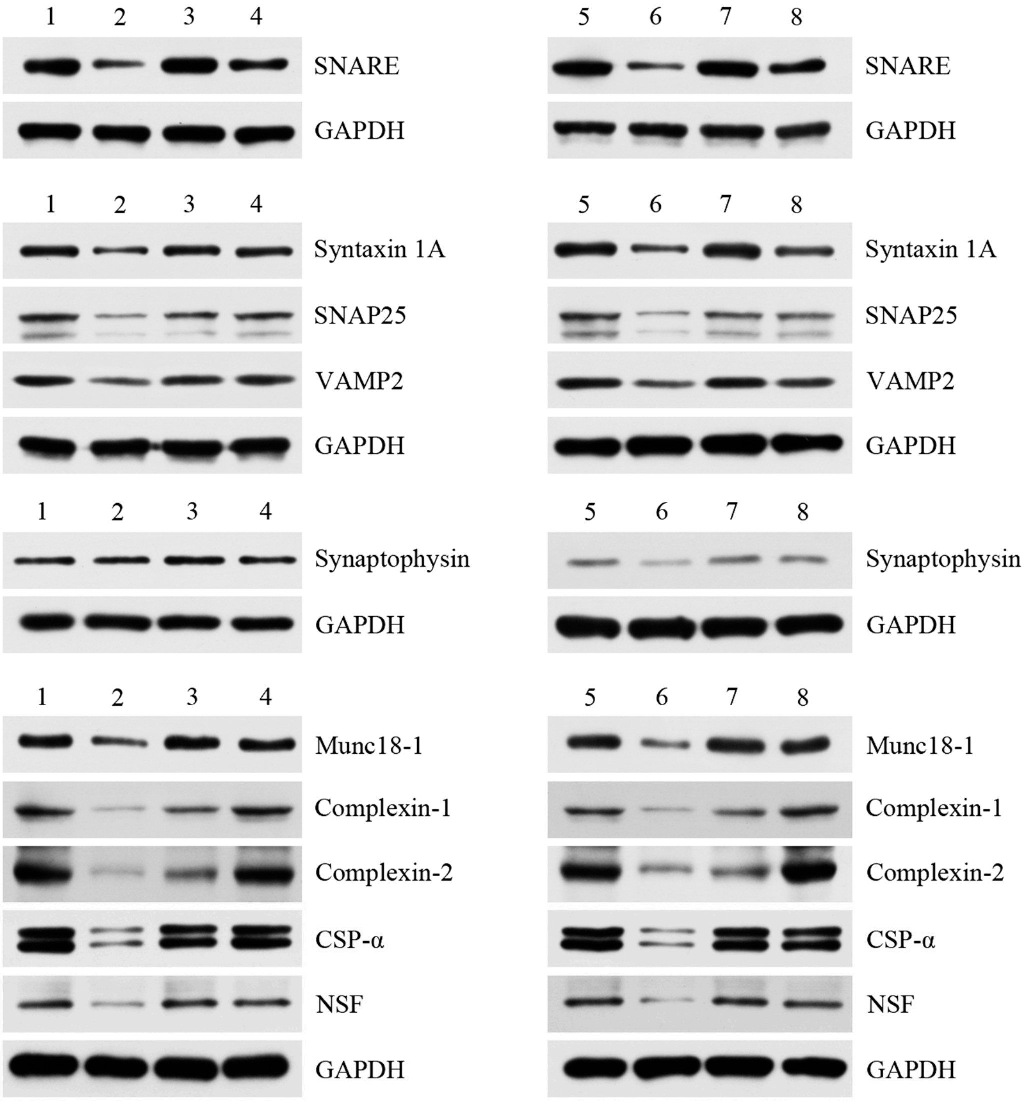


Figure S1 shows the original image of a western blot that was performed to detect the expression of a protein of interest in different samples. The image was captured using an imaging device and has not been edited or cropped.
